# Rates and causative pathogens of surgical site infections attributed to liver transplant procedures and other hepatic, biliary, or pancreatic procedures, 2015–2018

**DOI:** 10.1111/tid.13589

**Published:** 2021-03-23

**Authors:** Nora Chea, Mathew R. P. Sapiano, Liang Zhou, Lauren Epstein, Alice Guh, Jonathan R. Edwards, Katherine Allen-Bridson, Victoria Russo, Jennifer Watkins, Stephanie M. Pouch, Shelley S. Magill

**Affiliations:** 1 Division of Healthcare Quality Promotion, National Center for Emerging and Zoonotic Infectious Diseases, Centers for Disease Control and Prevention, Atlanta, GA, USA; 2 Lantana Consulting Group, Inc, East Thetford, VT, USA; 3 CACI Inc., Atlanta, GA, USA; 4 Emory University School of Medicine, Atlanta, GA, USA

**Keywords:** drug resistance, liver transplantation, microbial, surgical wound infection

## Abstract

Liver transplant recipients are at high risk for surgical site infections (SSIs). Limited data are available on SSI epidemiology following liver transplant procedures (LTPs). We analyzed data on SSIs from 2015 to 2018 reported to CDC’s National Healthcare Safety Network to determine rates, pathogen distribution, and antimicrobial resistance after LTPs and other hepatic, biliary, or pancreatic procedures (BILIs). LTP and BILI SSI rates were 5.7% and 5.9%, respectively. The odds of SSI after LTP were lower than after BILI (adjusted odds ratio = 0.70, 95% confidence interval 0.57–0.85). Among LTP SSIs, 43.1% were caused by *Enterococcus* spp., 17.2% by *Candida* spp., and 15.0% by coagulase-negative *Staphylococcus* spp. (CNS). Percentages of SSIs caused by *Enterococcus faecium* or CNS were higher after LTPs than BILIs, whereas percentages of SSIs caused by Enterobacteriaceae, *Enterococcus faecalis*, or viridans streptococci were higher after BILIs. Antimicrobial resistance was common in LTP SSI pathogens, including *E. faecium* (69.4% vancomycin resistant); *Escherichia coli* (68.8% fluoroquinolone non-susceptible and 44.7% extended spectrum cephalosporin [ESC] non-susceptible); and *Klebsiella pneumoniae* and *K. oxytoca* (39.4% fluoroquinolone non-susceptible and 54.5% ESC non-susceptible). National LTP SSI pathogen and resistance data can help prioritize studies to determine effective interventions to prevent SSIs and reduce antimicrobial resistance in liver transplant recipients.

## 1 | INTRODUCTION

Data from the Organ Procurement and Transplantation Network indicate that more than 165 000 liver transplants were performed in the United States from 1988 to 2018, of which 8250 were performed in 40 states, territories, and Washington, DC in 2018.^1^ Approximately 124 US centers performed at least one adult liver transplant procedure (LTP) between January 1 and December 31, 2018, according to the Scientific Registry of Transplant Recipients.^2^ Although survival rates among liver transplant recipients have improved significantly during recent decades,^3^ complications including surgical site infections (SSIs) remain common and are associated with significant morbidity and mortality.^4–8^

The risk for SSI among liver transplant recipients is heightened by multiple factors, including underlying comorbidities, complex transplant procedures involving the biliary tract, multiple transfusions of packed red blood cells, prolonged operative time, and intense immunosuppression.^4–14^ Limited data are available on LTP SSI rates, causal pathogens, and antimicrobial resistance, and most data available are from single centers. In these studies, SSI rates attributed to LTPs were reported to range from 8.8% to 37.8%, and the most common pathogens included Enterobacteriaceae, *Acinetobacter baumannii, Pseudomonas aeruginosa, Enterococcus* spp., *Staphylococcus aureus*, and *Candida* spp.^4,6,14,15^ To date, national data on SSIs attributed to LTPs in the United States have not been published. In addition, it is not clear how the characteristics of SSIs attributed to LTPs compare to those of SSIs attributed to non-transplant procedures involving the liver, biliary tract, or pancreas.

To prevent SSIs, the Clinical Practice Guidelines for Antimicrobial Prophylaxis in Surgery recommend perioperative prophylaxis with piperacillin-tazobactam or ampicillin plus cefotaxime for LTP.^16^ Guidelines published by the American Society of Transplantation Infectious Diseases Community of Practice are similar.^17^ However, antimicrobial prophylaxis recommendations are supported by limited evidence,^16^ and practices vary considerably among transplant centers.^18^ Many transplant centers also administer prophylactic antifungal medications perioperatively to high-risk recipients.^18–21^ Whether current surgical prophylaxis recommendations are consistent with national data on pathogens isolated from SSIs in liver transplant recipients is not known.

More than 6000 hospitals participate in surveillance for healthcare-associated infections, including SSIs, through the Centers for Disease Control and Prevention’s (CDC’s) National Healthcare Safety Network (NHSN).^22^ We analyzed NHSN surveillance data for SSIs following LTPs and other biliary tract, hepatic, or pancreatic procedures from January 1, 2015 through December 31, 2018 and reported to NHSN to determine rates, pathogen distributions, and antimicrobial resistance patterns.

## 2 | MATERIALS AND METHODS

### 2.1 | Setting and patient population

We analyzed data from hospitals conducting surveillance for SSIs attributed to LTP or other hepatic, biliary, or pancreatic procedures (the NHSN “BILI” operative procedure category) according to the NHSN protocol^23,24^ for at least 1 month from January 1, 2015 through December 31, 2018. The project was reviewed by a human subjects advisor of the CDC National Center for Emerging and Zoonotic Infectious Diseases and determined to be a non-research surveillance activity. According to the NHSN SSI protocol, patients who undergo LTPs or BILIs are monitored for 30 days following the procedure (where Day 1 = procedure date) for detection of SSIs. For each procedure under surveillance, the information reported includes, but is not limited to, age, gender, weight, wound class, operation duration, American Society of Anesthesiologists (ASA) score, and whether the operation was trauma related, performed using an endoscope (hereafter referred to as endoscopic surgery), or an emergency surgery. Patients’ diabetes mellitus status is also collected. If an SSI attributed to the procedure is identified, data collectors report the type of SSI (ie, superficial incisional, deep incisional, or organ/space) and whether infection was present at the time of surgery (PATOS).

Details of the NHSN SSI definitions are available online.^23^ In brief, superficial incisional SSIs involve only the skin and subcutaneous tissue, and are defined by the presence of purulent drainage from the superficial incision; identification of an organism from an aseptically obtained specimen from the superficial incision; selected signs and symptoms of infection in a patient whose incision is deliberately opened by a surgeon or other physician or physician designee but for whom no microbiological testing was performed; or diagnosis of superficial incisional SSI by a physician or physician designee. Deep incisional SSIs involve the deep soft tissues of the incision and are defined by purulent drainage from the deep incision; or spontaneous dehiscence or deliberate opening of the deep incision plus selected signs and symptoms of infection plus identification of an organism from the deep incision; or an abscess or other evidence of deep incisional infection detected on imaging or gross anatomical or histopathological examination. Organ/space SSIs involve tissues manipulated during the operative procedure that are deep to the fascia and muscle, and are defined by purulent drainage from a drain placed in the organ/space; identification of an organism from organ/space fluid or tissue; or an abscess or other evidence of organ/space infection detected on imaging or gross anatomical or histopathological examination. In addition, organ/space SSIs must meet additional criteria specific to the involved body site.^23^

Pathogen identification is a required element for meeting only some of the NHSN SSI definitions; however, if SSI-associated pathogens are identified, up to three pathogens can be reported for each infection event, along with selected antimicrobial susceptibility test (AST) results.^23^

### 2.2 | Antimicrobial resistance

Antimicrobial resistance profiles of pathogens reported for LTP or BILI SSIs were analyzed based on NHSN standard definitions.^25^ Extended-spectrum cephalosporin (ESC) non-susceptibility in *Escherichia coli* or *Klebsiella pneumoniae* and *K. oxytoca* (hereafter referred to as *K. pneumoniae/oxytoca*) was defined as an AST result of intermediate or resistant to cefepime, ceftazidime, cefotaxime, or ceftriaxone. Fluoroquinolone non-susceptibility in *E. coli* or *K. pneumoniae/oxytoca* was defined as an AST result of intermediate or resistant to ciprofloxacin, levofloxacin, or moxifloxacin. Carbapenem resistance in *E. coli* or *K. pneumoniae/oxytoca* was defined as an AST result of resistant to imipenem, meropenem, doripenem, or ertapenem. Multi-drug resistance in *E. coli* or *K. pneumoniae/oxytoca* was defined by non-susceptible AST results for at least one medication in three of the following antimicrobial groups: piperacillin or piperacillin/tazobactam (intermediate or resistant), ESCs (intermediate or resistant), fluoroquinolones (intermediate or resistant), aminoglycosides (intermediate or resistant to gentamicin, tobramycin, or amikacin), and carbapenems (resistant only).

### 2.3 | Descriptive and statistical analysis

We analyzed NHSN datasets generated on July 1, 2019, and excluded procedure and SSI records if they were submitted from long-term acute care hospitals or ambulatory surgery facilities, reported for outpatient procedures, or if infections involved a secondary incision site.

To describe LTP and BILLI SSI pathogen distributions and antimicrobial resistance, we included data from all hospitals reporting LTPs during the analysis period. Percentages of wound classes (clean or clean contaminated, contaminated, and dirty); ASA scores (1–5); male patients; emergency surgeries; endoscopic surgeries; median patient age; and median duration of surgery were calculated for LTPs and BILIs. SSI rates were calculated separately for LTPs and for BILIs as the number of SSIs divided by the number of procedures. Frequencies and distributions of SSI types (superficial incisional, deep incisional, and organ/space) and of SSIs attributed to common pathogens were also calculated for LTP and BILI SSIs. For each pathogen-antimicrobial combination with AST data available for ≥20 isolates, the percentage of resistant or non-susceptible pathogens was calculated as the number of resistant or non-susceptible pathogens divided by the number of pathogens with AST results reported to NHSN, multiplied by 100.

Among hospitals that reported both LTPs and BILIs, chi-squared tests were used to detect associations between procedure type (ie, LTP or BILI) and each causal pathogen, and between procedure type and resistance among specific pathogen-antimicrobial combinations. Fisher’s exact test was used for cases where the minimum cell size was <5.

Logistic regression was used to model factors affecting the odds of an SSI associated with an LTP or BILI procedure in hospitals performing both procedure types. To be consistent with the models developed by CDC to produce Standardized Infection Ratios,^26^ we chose the same variable categorization levels and restricted to patients ≥18 years. We excluded records for which selected variables were missing; if infection was reported to be PATOS; or if they were noted to be outliers for certain characteristics, including age, body mass index (BMI), and procedure duration. Stepwise selection was used to identify the final risk adjustment model (with an entry *P*-value of < .2 and a stay *P*-value of < .05), with procedure type excluded from this step. A binary variable denoting procedure type (LTP or BILI) was then introduced to determine whether there was a statistically significant difference in the SSI rate between the two procedures after accounting for the risk factors.

A second risk adjustment model was developed using the same exclusions and stepwise process to test for a difference in LTP SSI rates between the time periods 2015–2016 and 2017–2018 in all hospitals performing LTPs, using a binary variable denoting the 2-year time period during which the procedure occurred. *P*-values < .05 were considered statistically significant. Analysis was generated using SAS software version 9.4 (SAS Institute Inc).

## 3 | RESULTS

### 3.1 | Hospitals, procedures, and SSI rates

During 2015–2018, a total of 74 818 LTPs and BILIs were reported to NHSN. After excluding incomplete records, outpatient procedures, and procedures performed in ambulatory surgery centers or long-term acute care hospitals, 54 653 procedures remained: 47 454 BILIs from 403 hospitals, and 7199 LTPs from 44 hospitals. Of the 44 hospitals reporting LTPs, 30 also reported ≥1 BILI. Most hospitals reporting LTPs and BILIs were general teaching hospitals with ≥200 beds (Table 1).

Characteristics of LTPs in all 44 hospitals and BILIs in 30 hospitals also performing LTPs are shown in Table 2. The median duration of LTPs was longer than the median duration of BILIs; ASA scores were higher for LTPs; and a larger percentage of LTPs were reported as emergency surgeries. Most SSIs attributed to LTPs and BILIs were organ/space infections with ≥1 pathogen reported. Of the 44 hospitals performing LTPs, 30 (68.2%) reported ≥1 LTP-associated SSI, with a total of 413 SSIs among 7199 LTPs (5.7%) and hospital-specific SSI rates that ranged from 0% to 15.9%. Of the 30 hospitals performing both LTPs and BILIs, 20 hospitals (66.7%) reported ≥1 LTP-associated SSI and 26 hospitals (86.7%) reported ≥1 BILI-associated SSI. In these 30 hospitals, there were 280 SSIs among 5206 LTPs (5.4%) and 1136 SSIs among 19 317 BILIs (5.9%); hospital-specific SSI rates ranged from 0% to 9.4% after LTPs and 0 to 17.1% after BILIs.

In a multivariable logistic regression model evaluating risk factors for SSI among adult patients undergoing LTPs or BILIs in the same group of hospitals, the odds of SSI after LTPs were significantly lower than after BILIs (odds ratio [OR] = 0.70, 95% confidence interval [CI] 0.57–0.85), after adjusting for procedure duration, ASA score, gender, surgery type (endoscopic vs non-endoscopic; emergency vs non-emergency surgeries), and age (Table 3).

Among hospitals performing LTPs, we observed a statistically significant decrease in pooled mean LTP SSI rate from the first 2 years (2015–2016, 6.1%) to the last 2 years (2017–2018, 4.6%) of the analysis period after adjusting for other factors (OR = 0.73, 95% CI 0.59–0.91; Table 4).

### 3.2 | Pathogens isolated from SSIs

A total of 560 pathogens were reported for 387 of 413 LTP SSIs (93.7%); one pathogen was reported for 256 SSIs (62.0% of all SSIs), two pathogens for 89 SSIs (21.5%), and three pathogens for 42 SSIs (10.2%). Of all LTP SSIs reported from the 44 hospitals, 178 (43.1%) were caused by *Enterococcus* spp. (including *Enterococcus faecium* and *Enterococcus faecalis*), 71 (17.2%) by *Candida* spp. (including *Candida albicans* and *Candida glabrata*), 62 (15.0%) by coagulase-negative *Staphylococcus* spp. (CNS), 50 (12.1%) by *E. coli*, 39 (9.4%) by *Staphylococcus aureus*, and 35 (8.5%) by *K. pneumoniae/oxytoca* (Table 5). *Enterococcus* spp. (including *E. faecium* and *E. faecalis*) were commonly reported as causes of organ/space infections (154/319, 48.3%), whereas *Staphylococcus* spp. (including *S. aureus*) were commonly reported for superficial or deep incisional infections (45/94, 47.9%) (Table 6).

Although the most common LTP SSI pathogens overall were similar when compared with BILI SSI pathogens, the percentages of SSIs caused by selected pathogen groups differed significantly (Table 5). For example, the percentage of SSIs caused by *E. faecium* or CNS was significantly higher after LTPs than after BILIs, whereas the percentages of SSIs caused by Enterobacteriaceae (including *E. coli*, *K. pneumoniae/oxytoca, Enterobacter* spp., and *Citrobacter* spp.), *E. faecalis*, or viridans streptococci were significantly higher after BILIs.

### 3.3 | Antimicrobial resistance

Susceptibility results for antibiotics of interest were available for more than 90% of selected common LTP and BILI SSI pathogens (Table 7). Overall, the prevalence of antibiotic resistance was substantially higher among pathogens isolated from LTP SSIs than among pathogens isolated from BILI SSIs. High percentages of resistance phenotypes were noted in *E. faecium* (69.4% vancomycin-resistant, VRE), *E. coli* (68.8% fluoroquinolone non-susceptible and 44.7% ESC non-susceptible), and *K. pneumoniae/oxytoca* (39.4% fluoroquinolone non-susceptible and 54.5% ESC non-susceptible) isolated from LTP SSIs. Carbapenem resistance among selected Enterobacteriaceae was uncommon but was significantly more prevalent among pathogens isolated from LTP SSIs than BILI SSIs. Similarly, the percentage of *E. coli* and *K. pneumoniae/oxytoca* that were multidrug-resistant (MDR) among LTP SSIs was significantly higher than among BILI SSIs.

## 4 | DISCUSSION

This is the first report of the pathogens isolated and the frequency of select antimicrobial resistance phenotypes from SSIs attributed to LTPs and BILIs from multiple US hospitals nationwide. Based on data from the Scientific Registry of Transplant Recipients,^2^ the hospitals contributing data to this analysis represent approximately one third of hospitals performing liver transplants in the United States, making it among the largest analyses of LTP SSIs to date. In our analysis, the pooled mean rate of SSIs attributed to LTPs was lower than previously reported by others, and we observed a significantly lower LTP SSI rate in 2017–2018 compared with 2015–2016. Despite this evidence of progress in SSI prevention among liver transplant recipients, our data also show that antimicrobial resistance is a significant concern among SSIs following LTP, particularly when contrasted with resistance in pathogens isolated from SSIs in patients undergoing other surgical procedures involving similar anatomical sites.

We observed a pooled mean LTP SSI rate of 5.7%, with a range of 0%−15.9% among hospitals included in our analysis. The rate we observed is lower than rates reported in studies of LTPs performed from 2010 to 2014^4^ or 2011 to 2014,^5^ which ranged from 8.8% to 37.8%. Improvements in surgical technique and perioperative infection prevention practices since the time period covered by these earlier studies could explain the lower SSI rates we observed. In addition, the lower rate in our analysis could be partially explained by differences in surveillance methods, including a shorter follow-up period (eg, 30 days vs 60 or 90 days) and surveillance definitions. For example, a study by Viehman et al in which liver transplant recipients were followed for 90 days after surgery showed a SSI rate of 18%.^4^ The rate we observed is also lower than the rate reported in a more recent analysis of data from the National Surgical Quality Improvement Project’s transplant initiative (NSQIP Transplant); in this study of 1048 liver transplant recipients in 2017–2018, the crude SSI rate within 30 days of LTP was 9.7%.^27^ As in our analysis, the NSQIP Transplant investigators observed substantial variability among centers, with SSI rates from 0% to 29%.^27^ Other studies have shown discrepancies between SSI rates determined through NSQIP and NHSN, likely as a result of differences in surveillance methods between the two systems.^28,29^

We found that after adjusting for other factors, LTPs were associated with lower odds of SSI compared to BILIs. This finding is somewhat surprising, given the surgical complexity of LTPs and recipient immuno-suppression, but could be explained in part by differences in the array of operative procedures or underlying conditions of patients receiving BILIs vs LTPs, or by differences in perioperative antimicrobial prophylaxis or other infection prevention practices.^4,5,14,18^ Other studies have shown that SSI rates after non-transplant hepatic, pancreatic, or complex biliary surgeries tend to be relatively high despite perioperative antimicrobial prophylaxis.^30–32^ For example, Ceppa et al showed that SSI rates for these procedures ranged from approximately 24% to 28% before interventions and 11% to 17% after interventions which included perioperative antimicrobial prophylaxis.^30^ Although some have shown there is no difference in hand hygiene compliance among providers caring for transplant patients vs other patients,^33^ it is possible that there are differences in adherence to other SSI prevention measures. Only limited data are available on adherence to skin preparation recommendations, glycemic control, and maintenance of normothermia during LTP and other procedures.^34^

Recent data on pathogens isolated from SSIs in liver transplant recipients are also limited, with no large, multicenter studies to which we can compare our results. SSI prevention guidelines from the Transplant Infectious Diseases Community of Practice identify selected gram-negative bacteria, including Enterobacteriaceae, *Acinetobacter*, and *Pseudomonas*, enterococci, staphylococci, and *Candida* species as pathogens of concern after liver transplantation.^17^ We observed that *Pseudomonas aeruginosa* was infrequently reported as an SSI pathogen following LTP (3.6% of SSIs), and only one *Acinetobacter* infection was reported. In a single-center study of LTPs performed from 2010 to 2014 at the University of Pittsburgh, *Enterococcus* spp., Enterobacteriaceae, and *Candida* spp. were common causes of deep SSIs within 90 days of surgery; as in our analysis, *E. faecium* was the single most common pathogen isolated. Antimicrobial resistance was prevalent, and 82% of patients with deep SSIs were infected with pathogens that were not susceptible to the antimicrobial medications given for perioperative prophylaxis.^4^

Perioperative antimicrobial prophylaxis remains a principal component of SSI prevention strategies for LTPs despite the absence of clinical trials demonstrating efficacy and limited data available for guiding antimicrobial selection.^17^ Whether broad-spectrum antimicrobial prophylaxis is better than narrow-spectrum coverage is not clear.^13,14^ Our analysis showed that 20.3% of all LTP SSIs were caused by vancomycin-resistant *E. faecium* and 9.4% by ESC-resistant *E. coli* or *K. pneumoniae/oxytoca*, raising the concern that current prophylaxis recommendations, which include third-generation cephalosporins, may be selecting for these resistant pathogens. Antimicrobial exposure is common in patients with advanced liver disease, who are at high risk for serious infections, and studies have shown that substantial percentages of patients are colonized with VRE and other resistant pathogens before transplantation.^35–39^ Some centers screen patients pre-transplant to detect colonization with resistant organisms and tailor perioperative prophylaxis.^40^ Other approaches to prevent colonization and infection of patients before and after transplantation are needed. These include antimicrobial stewardship and infection control interventions in the peritransplant period^41^; increased focus on improving antimicrobial use among patients with liver disease who are expected to require transplantation in the future may also be warranted.

Our analysis has limitations. Reporting of LTP and BILI SSI surveillance data to NHSN is voluntary, except for in California and Pennsylvania. Our pooled mean SSI rate is based on data submitted by a subset of hospitals performing LTPs in the United States, and therefore our results may not be generalizable to all hospitals. In addition, because of limited reporting we were not able to describe antifungal resistance among *Candida* spp., the second most common pathogen group overall isolated from LTP SSIs. Finally, information on perioperative antimicrobial prophylaxis practices in the participating hospitals was not available. This information may have allowed us to have a better understanding of the pathogen distribution and antibiotic resistance patterns we observed.

## 5 | CONCLUSION

Our analysis showed that the pooled mean SSI rate attributed to LTPs was lower than previously reported and improved from 2015–2016 to 2017–2018. The odds of SSI after LTP were significantly lower than following other procedures involving similar anatomical sites, after adjusting for other factors. Most LTP SSIs were organ/space infections and caused by pathogens with high levels of antibiotic resistance. Recommended antimicrobial regimens for perioperative prophylaxis may not cover some pathogens commonly associated with LTP SSIs. National LTP SSI pathogen and antimicrobial resistance data can help prioritize studies to determine effective interventions to prevent SSIs and reduce antimicrobial resistance in liver transplant recipients, including approaches to optimize antimicrobial prescribing for perioperative prophylaxis and empiric treatment of SSIs in the early postoperative period.

## Figures and Tables

**TABLE 1:** Characteristics of hospitals reporting surgical site infections (SSIs) attributed to liver transplant procedures (LTPs) or other hepatic, biliary, or pancreatic procedures (BILIs), National Healthcare Safety Network, 2015–2018

Characteristic	No. of hospitals reporting LTPs (%) N = 44	No. of hospitals reporting both LTPs and BILIs (%) N = 30^a^
Hospital type		
General hospital	38 (86.4)	25 (83.3)
Children’s hospital	5 (11.4)	4 (13.3)
Other	1 (2.3)	1 (3.3)
Census region		
Northeast	5 (11.4)	5 (16.7)
Midwest	10 (22.7)	3 (10.0)
South	5 (11.4)	2 (6.7)
West	24 (54.5)	20 (66.7)
Hospital size, no. beds		
51–200	1 (2.3)	1 (3.3)
201–500	28 (63.6)	20 (66.7)
≥501	15 (34.1)	9 (30.0)
Academic affiliation		
Teaching	41 (93.2)	28 (93.3)
Non-teaching	3 (6.8)	2 (6.7)

a 14 hospitals of 44 total hospitals reporting LTPs did not report any BILIs from 2015 to 2018.

**TABLE 2:** Characteristics of liver transplant procedures (LTPs) or other hepatic, biliary, or pancreatic procedures (BILIs), National Healthcare Safety Network, 2015–2018

Characteristic	LTPs	BILIs
No. hospitals reporting data	44	30^a^
Total no. procedures reported	7199	19 317
Wound class - no. (%)		
Clean or clean contaminated^b^	7022 (97.5)	17 640 (91.3)
Contaminated	135 (1.9)	1191 (6.2)
Dirty	42 (0.6)	486 (2.5)
Male - no. (%)	4575 (63.6)	9688 (50.2)
Median age in years (IQR)	57 (47–63)	60 (47–69)
Endoscopic surgery - no. (%)^c^	--	4256 (22.0)
Median duration in minutes (IQR)	359 (279–457)	233 (140–350)
ASA score - no. (%)		
1	12 (0.2)	536 (2.8)
2	62 (0.9)	4967 (25.7)
3	1772 (24.6)	11 570 (59.9)
4	5201 (72.2)	2096 (10.9)
5	152 (2.1)	148 (0.8)
Emergency surgery - no. (%)	3452 (48.0)	2400 (12.4)
Total no. surgical site infections	413	1136
Superficial incisional - no. (%)	78 (18.9)	235 (20.7)
Deep incisional - no. (%)	16 (3.9)	68 (6.0)
Organ/space - no. (%)	319 (77.2)	833 (73.3)
Infection present at time of surgery (PATOS) - no. (%)	18 (4.4)	66 (5.8)
Infections with ≥1 pathogen reported - no. (%)	387 (93.7)	989 (87.1)

Abbreviations: ASA, American Society of Anesthesiologists; IQR, interquartile range.

a 14 hospitals of 44 total hospitals reporting LTPs did not report any BILIs from 2015 to 2018.

b 1217 LTPs were reported as clean procedures. BILIs cannot be reported to NHSN as clean procedures.

c 22 LTPs were reported as endoscopic surgery. According to the NHSN surveillance protocol, LTPs should not be reported as endoscopic surgery. We reassigned these 22 LTPs as non-endoscopic surgery for this analysis.

**TABLE 3:** Multivariable logistic regression model^a^ to identify factors associated with surgical site infections (SSIs) among 22 977^b^ liver transplant procedures (LTPs) or other hepatic, biliary, or pancreatic procedures (BILIs) in 30 hospitals performing both procedure types and reporting to the National Healthcare Safety Network (NHSN), 2015–2018

Variable	No. of procedures	No. of procedures with SSI (%)	Odds ratio (95% CI)	*P*-value
Procedure duration quartile				
1st quartile (<163 min)	5672	137 (2.4)	Ref	--
2nd quartile (163–269 min)	5773	275 (4.8)	2.00 (1.62–2.47)	<.001
3rd quartile (270–387 min)	5757	381 (6.6)	3.04 (2.48–3.73)	<.001
4th quartile (≥388 min)	5775	491 (8.5)	4.16 (3.40–5.10)	<.001
ASA score				
1	490	10 (2.0)	Ref	--
2	4781	257 (5.4)	3.27 (1.72–6.23)	<.001
3	12 050	758 (6.3)	3.43 (1.81–6.51)	<.001
4	5448	250 (4.6)	2.65 (1.37–5.12)	0.004
5	208	9 (4.3)	3.32 (1.31–8.42)	0.012
Gender				
Female	10 760	520 (4.8)	Ref	--
Male	12 217	764 (6.3)	1.25 (1.12–1.41)	<.001
Emergency procedure				
Non-emergency surgery	18 019	1073 (6.0)	Ref	--
Emergency surgery	4958	211 (4.3)	0.82 (0.69–0.98)	.028
Patient age quartile				
1st quartile (<50 y)	5498	271 (4.9)	Ref	--
2nd quartile (50–59 y)	5409	275 (5.1)	0.93 (0.78–1.11)	.418
3rd quartile (60–67 y)	5928	342 (5.8)	1.05 (0.89–1.24)	.585
4th quartile (≥68 y)	6142	396 (6.4)	1.16 (0.98–1.37)	.075
Endoscopic approach^c^				
Endoscopic surgery	4061	163 (4.0)	Ref	--
Non-endoscopic surgery	18 916	1121 (5.9)	1.24 (1.04–1.48)	.017
Procedure type				
Other biliary, hepatic, or pancreatic	18 214	1050 (5.8)	Ref	--
Liver transplant	4763	234 (4.9)	0.70 (0.57–0.85)	<.001

Abbreviations: ASA, American Society of Anesthesiologists; CI, confidence interval.

a Other variables that were tested but found not to be statistically significant predictors of SSI risk were diabetes, incision closure type, wound class, trauma, hospital teaching status, body mass index category, and hospital bed size category.

b Model excludes procedures in patients <18 or >109 years old; patients with BMI <12 or BMI >60; with infection PATOS; reported to have an implausibly short or long procedure duration; or with missing incision closure type, wound class, ASA score, or patient gender.

c 12 LTPs were reported as endoscopic surgery. According to the NHSN surveillance protocol, LTPs should not be reported as endoscopic surgery. We reassigned these 12 LTPs as non-endoscopic surgery for this analysis.

**TABLE 4:** Multivariable logistic regression model^a^ to identify factors associated with surgical site infections (SSIs) among 6653^b^ liver transplant procedures (LTPs) reported to the National Healthcare Safety Network (NHSN), 2015–2018

Variable	No. of procedures	No. of procedures with SSI (%)	Odds ratio (95% CI)	*P*-value
Emergency procedure				
Non-emergency surgery	3411	209 (6.1)	Ref	--
Emergency surgery	3242	144 (4.4)	0.71 (0.57–0.88)	.002
Patient age quartile				
1st quartile (<50 y)	1585	100 (6.3)	Ref	--
2nd quartile (50–59 y)	2124	121 (5.7)	0.87 (0.66–1.14)	.315
3rd quartile (60–67 y)	2288	110 (4.8)	0.71 (0.54–0.95)	.020
4th quartile (≥68 y)	656	22 (3.4)	0.51 (0.32–0.82)	.006
Diabetes				
No	4741	237 (5.0)	Ref	--
Yes	1912	116 (6.1)	1.32 (1.05–1.67)	.019
Incision closure				
Primary	6598	346 (5.2)	Ref	--
Non-primary	55	7 (12.7)	2.43 (1.09–5.44)	.031
Time period				
2015–2016	2978	182 (6.1)	Ref	--
2017–2018	3675	171 (4.7)	0.73 (0.59–0.91)	.005

Abbreviations: ASA, American Society of Anesthesiologists; CI, confidence interval.

a Other variables that were tested but found not to be statistically significant predictors of LTP SSI risk were gender, ASA score category, wound class, procedure duration quartile, trauma, hospital teaching status, body mass index category, and hospital bed size category.

b Model includes data from 41/44 hospitals performing LTPs. 546 LTPs (with 59 SSIs) were excluded due to patient age <18 or >109 years old; patient BMI <12 or BMI >60; with infection PATOS; reported to have an implausibly short or long procedure duration; or with missing incision closure type, wound class, ASA score, or patient gender.

**TABLE 5:** Pathogen distribution among surgical site infections (SSIs) attributed to liver transplant procedures (LTPs) or other hepatic, biliary, pancreatic procedures (BILIs), National Healthcare Safety Network, 2015–2018

Pathogen	All 44 hospitals reporting LTPs	30 hospitals reporting both LTPs and BILIs	
	No. of LTP SSIs (%), N = 413^a^	No. of LTP SSIs (%), N = 280^b^	No. of BILI SSIs (%), N = 1136^c^
*Enterococcus faecium*	124 (30.0)	80 (28.6)^d^	86 (7.6)
Coagulase-negative *Staphylococcus* spp.	62 (15.0)^e^	45 (16.1)^d^	91 (8.0)^f^
*Escherichia coli*	50 (12.1)	26 (9.3)^d^	191 (16.8)
*Staphylococcus aureus*	39 (9.4)	27 (9.6)	108 (9.5)
*Klebsiella pneumoniae/oxytoca*	35 (8.5)	29 (10.4)^d^	180 (15.8)^g^
*Enterococcus faecalis*	33 (8.0)	15 (5.4)^d^	131 (11.5)
Other *Enterococcus* spp.	33 (8.0)^h^	25 (8.9)	98 (8.6)^i^
*Candida albicans*	28 (6.8)	18 (6.4)^d^	119 (10.5)
*Candida glabrata*	28 (6.8)	22 (7.9)^d^	42 (3.7)
Other *Candida* spp.	19 (4.6)	16 (5.7)	44 (3.9)
*Pseudomonas aeruginosa*	15 (3.6)	11 (3.9)	33 (2.9)
Viridans streptococci	14 (3.4)	8 (2.9)^d^	108 (9.5) ^j^
*Enterobacter cloacae* complex	12 (2.9)	7 (2.5)^d^	125 (11.0)
*Lactobacillus* spp.	9 (2.2)	6 (2.1)	25 (2.2)
*Stenotrophomonas maltophilia*	6 (1.5)	3 (1.1)	5 (0.4)
*Bacteroides* spp.	5 (1.2)	2 (0.7)^d^	33 (2.9)
Other *Enterobacter* spp.^k^	5 (1.2)	2 (0.7)^d^	37 (3.3)
*Citrobacter* spp.	0	0^d^	37 (3.3)
Other	40 (9.7)^l^	29 (10.4)^d^	197 (17.3)^m^
No pathogen reported	26 (6.3)	19 (6.8)^d^	147 (12.9)

a Numbers sum to >100 percent because multiple pathogens were reported for some SSIs. A total of 560 pathogens were reported for 387/413 LTP SSIs (93.7%).

b Numbers sum to >100 percent because multiple pathogens were reported for some SSIs. A total of 371 pathogens were reported for 261/280 LTP SSIs (93.2%).

c Numbers sum to >100 percent because multiple pathogens were reported for some SSIs. A total of 1726 pathogens were reported for 989/1136 BILI SSIs (87.0%).

d Denotes a statistically significant difference when compared to the pathogen percentage in BILI SSIs.

e 63 pathogens were reported for 62 SSIs; 1 SSI had 2 coagulase-negative staphylococci reported.

f 93 pathogens were reported for 91 SSIs; 2 SSIs each had 2 coagulase-negative staphylococci reported.

g 182 pathogens were reported for 180 SSIs; 2 SSIs each had *Klebsiella pneumoniae* and *Klebsiella oxytoca* reported.

h 34 pathogens were reported for 33 SSIs; 1 SSI had 2 *Enterococcus* spp. reported.

i 99 pathogens were reported for 98 SSIs; 1 SSI had 2 *Enterococcus* spp. reported.

j 110 pathogens were reported for 108 SSIs; 2 SSIs each had 2 viridans streptococci reported.

k *Klebsiella aerogenes* (formerly *Enterobacter aerogenes*) was included with other *Enterobacter* spp. for this analysis.

l 41 pathogens were reported for 40 SSIs. Other pathogens included yeast not otherwise specified (4), *Corynebacterium* spp. (3), *Klebsiella* spp. other than *pneumoniae* or *oxytoca* (3), *Saccharomyces cerevisiae* (3), *Serratia marcescens* (3), *Streptococcus* spp. (3), *Clostridium perfringens* (2), fungus not otherwise specified (2), *Morganella morganii* (2), *Acinetobacter* spp. (1), *Aspergillus fumigatus* (1), *Bacillus cereus* (1), *Burkholderia cepacia* (1), diphtheroids (1), *Finegoldia* spp. (1), *Fusarium* spp. (1), gram-positive cocci not otherwise specified (1), group B *Streptococcus* (1), *Hafnia alvei* (1), *Peptostreptococcus* spp. (1), *Pseudomonas fluorescens* (1), *Proteus mirabilis* (1), *Raoultella* spp. (1), *Rhizopus* spp. (1), and *Weissella confusa* (1).

m 226 pathogens were reported for 197 SSIs.

**TABLE 6:** Pathogen distribution among surgical site infections (SSIs) attributed to liver transplant procedures (LTPs), stratified by SSI type, National Healthcare Safety Network, 2015–2018

Pathogen	Surgical site infection type	
	No. of organ/space infections (%), N = 319^a^	No. of superficial or deep incisional infections (%), N = 94^b^
*Enterococcus faecium*	112 (35.1)	12 (12.8)
Coagulase-negative *Staphylococcus* spp.	41 (12.9)	21 (22.3)^c^
*Escherichia coli*	45 (14.1)	5 (5.3)
*Staphylococcus aureus*	14 (4.4)	25 (26.6)
*Klebsiella pneumoniae/oxytoca*	31 (9.7)	4 (4.3)
*Enterococcus faecalis*	30 (9.4)	3 (3.2)
Other *Enterococcus* spp.	28 (8.8)^d^	5 (5.3)
*Candida albicans*	28 (8.8)	0
*Candida glabrata*	27 (8.5)	1 (1.1)
Other *Candida* spp.	18 (5.6)	1 (1.1)
*Pseudomonas aeruginosa*	11 (3.4)	4 (4.3)
Viridans streptococci	13 (4.1)	1 (1.1)
*Enterobacter cloacae* complex	12 (3.8)	0
*Lactobacillus* spp.	8 (2.5)	1 (1.1)
*Stenotrophomonas maltophilia*	6 (1.9)	0
*Bacteroides* spp.	5 (1.6)	0
Other *Enterobacter* spp.^e^	5 (1.6)	0
Other pathogens	34 (10.7)^f^	6 (6.4)
No pathogen reported	7 (2.2)	19 (20.2)

a Numbers sum to >100 percent because multiple pathogens were reported for some SSIs. 470 total pathogens reported for 312/319 organ/space surgical site infections.

b Numbers sum to >100 percent because multiple pathogens were reported for some SSIs. 90 total pathogens reported for 75/94 superficial or deep incisional surgical site infections.

c 22 pathogens reported for 21 SSIs; 1 SSI had 2 coagulase-negative staphylococci reported.

d 29 pathogens reported for 28 SSIs; 1 SSI had 2 *Enterococcus* spp. reported.

e *Klebsiella aerogenes* (formerly *Enterobacter aerogenes*) was included with other *Enterobacter* spp. for this analysis.

f 35 pathogens reported for 34 SSIs; 1 SSI had 2 other pathogens reported.

**TABLE 7:** Antimicrobial resistance among pathogens from surgical site infections (SSIs) attributed to liver transplant procedures (LTPs) or other hepatic, biliary, or pancreatic procedures (BILIs), National Healthcare Safety Network, 2015–2018

Pathogen and antimicrobial resistance phenotype	All 44 hospitals reporting LTPs		30 hospitals reporting LTPs and BILIs			
	LTP SSI No. of isolates tested (of no. reported)	LTP SSI No. resistant or non-susceptible (%)	LTP SSI No. of isolates tested (of no. reported)	LTP SSI No. resistant or non-susceptible (%)	BILI SSI No. of isolates tested (of no. reported)	BILI SSI No. resistant or non-susceptible (%)
*Staphylococcus aureus*						
Oxacillin, methicillin, or cefoxitin	36 (of 39)	19 (52.8)	24 (of 27)	13 (54.2)	102 (of 108)	34 (33.3)
*Enterococcus faecium*						
Vancomycin	121 (of 124)	84 (69.4)	79 (of 80)	60 (75.9)^a^	83 (of 86)	42 (50.6)
*Enterococcus faecalis*						
Vancomycin	32 (of 33)	1 (3.1)	14 (of 15)	--^b^	120 (of 131)	3 (2.5)
*Escherichia coli*						
Fluoroquinolone	48 (of 50)	33 (68.8)	25 (of 26)	16 (64.0)^a^	181 (of 191)	71 (39.2)
ESC	47 (of 50)	21 (44.7)	23 (of 26)	13 (56.5)^a^	172 (of 191)	47 (27.3)
Carbapenems	46 (of 50)	3 (6.5)	22 (of 26)	1 (4.5)	158 (of 191)	1 (0.6)
MDR	48 (of 50)	15 (31.3)	24 (of 26)	8 (33.3)^a^	183 (of 191)	28 (15.3)
*Klebsiella pneumoniae/oxytoca*						
Fluoroquinolone	33 (of 35)	13 (39.4)	28 (of 29)	13 (46.4)^a^	171 (of 180)	15 (8.8)
ESC	33 (of 35)	18 (54.5)	28 (of 29)	16 (57.1)^a^	159 (of 180)	25 (15.7)
Carbapenems	29 (of 35)	3 (10.3)	24 (of 29)	3 (12.5)^a^	143 (of 180)	3 (2.1)
MDR	33 (of 35)	11 (33.3)	28 (of 29)	10 (35.7)^a^	173 (of 180)	11 (6.4)

Abbreviations: ESC, extended-spectrum cephalosporin non-susceptibility; Fluoroquinolone, fluoroquinolone non-susceptibility; MDR, multi-drug resistant; Oxacillin, methicillin, or cefoxitin, oxacillin or methicillin or cefoxitin resistance; Vancomycin, vancomycin resistance.

a Denotes a statistically significant difference when compared to the pathogen percentage in BILI SSIs.

b Too few isolates tested to report (<20).

## Abbreviations:

ASA: American Society of Anesthesiologists
AST: antimicrobial susceptibility testing
BILI: bile duct, liver, or pancreatic surgery
CDC: Centers for Disease Control and Prevention
CI: confidence interval
CNS: coagulase-negative *Staphylococcus* spp.
ESC: extended-spectrum cephalosporin
IQR: interquartile range
LTP: liver transplant procedure
MDR: multidrug-resistant
NHSN: National Healthcare Safety Network
PATOS: infection present at time of surgery (PATOS)
SSI: surgical site infection
VRE: vancomycin-resistant *Enterococcus*
